# Sepsis: New Challenges and Future Perspectives for an Evolving Disease—Precision Medicine Is the Way!

**DOI:** 10.3390/medicina57101109

**Published:** 2021-10-15

**Authors:** Antonio Mirijello, Alberto Tosoni

**Affiliations:** 1 Internal Medicine Unit, Department of Medical Sciences, IRCCS Casa Sollievo della Sofferenza, 71013 San Giovanni Rotondo, Italy; 2 Department of Internal Medicine and Gastroenterology, Fondazione Policlinico Universitario “A. Gemelli” IRCCS, 00168 Rome, Italy

Sepsis still remains the leading cause of in-hospital death in the world [1,2]. In the very recent years, this condition became even more evident with the appearance of COVID-19, which has reached an unexpected burden in terms of morbidity and mortality worldwide [3]; the severe form of COVID-19 is characterized by multi-organ failure, mainly secondary to an inappropriate host response, which can be considered a full-fledged sepsis [4]. In addition, SARS-CoV-2 infection has been shown to be an independent risk factor for the development of bloodstream infection (BSI) and sepsis in hospitalized patients [5].

The knowledge in the field of sepsis has accumulated over time. Nevertheless, significant gaps in understanding the pathophysiological aspects and the management possibilities of this deadly condition are still present.

The Special Issue “New Strategies for Treatment of Sepsis” was thought as an opportunity to bring together high-quality manuscripts that showcase the current knowledge on the management of sepsis. We were particularly aware that several unmet needs still needed to be addressed: the comprehension of mechanisms underlying the development and progression of sepsis, the use of new diagnostic tools (including artificial intelligence) for a better and less invasive approach, and the development of antimicrobial strategies in order to effectively fight antimicrobial resistance [1].

The findings reported in the studies published as part of this Special Issue further confirm the potential beneficial role of a deep understanding of mechanisms underlying sepsis, both in reference to the characteristics of microbes and of hosts involved, which in their singularity go to outline different types of sepsis each time. In this way this Special Issue pave the way for future investigations aimed at further dissecting the impact of Precision Medicine as the leading strategy for treatment of sepsis.

Specifically, the article by Rossetti et al. comprehensively analyzed the substantial changes in the homeostasis of micronutrients connected with sepsis and its regulatory processes [6]. In particular, the roles of Vitamin D, Vitamin C, Thiamine and Zinc, all involved in inflammatory or immune response processes, were analyzed. Authors reviewed several studies, many of which have failed to achieve statistical significance or contradict each other. However, the main limitation of these studies, having mortality as outcome, is that the action of micronutrients on seriously ill patients may be less relevant, probably because the severity of organ failure is the result of several metabolic pathways that cannot be easily reverted. Research on this topic should consider reliable and clinically sensitive surrogate outcomes besides mortality, since in seriously ill patients the outcome is too often confused by concomitant factors.

On this connection, the article by Aisa-Alvarez et al. highlighted the antioxidant effect of Vitamin C, Vitamin E, N-acetylcysteine, and Melatonin in patients with septic shock determining the SOFA score and measuring antioxidant markers in plasma [7]. Other secondary outcomes were measured on day 28th including mortality due to any cause, ventilator-free days, ICU-free days, and hospital-free days. The results showed that antioxidant therapy associated with standard therapy reduces multi-organ failure, oxidative stress, and inflammation in patients with septic shock. The strengths of this study are certainly the monitoring of the plasma levels of the micronutrients administered, the evident paucity of treatment-associated side effects, a rapid assessment of patients that allowed to start the administration of micronutrients in a much faster way compared to other comparable studies. Moreover, this is the first study in which the use of Melatonin has been tested in humans with septic shock.

Back to pathophysiological aspects of sepsis, the study by Cutuli et al. analyzed the possibility of both pharmacological and extracorporeal immune modulation in critically ill septic patients [8]. In the last few years, an increasing body of evidence has demonstrated that the administration of immune modulating drugs can mitigate both pro- and anti-inflammatory bursts due to an infection and should be considered as a complementary therapy to be associated with appropriate etiologic therapies (e.g., source infection control and antibiotics). However, the real application of this complementary treatment is still a matter of debate due to controversial results between laboratory and clinical trials. Trials may be inconclusive or discordant with each other due to the great heterogeneity of septic patients enrolled [9]. It is mandatory to push towards a personalized approach trying to distinguish the peculiar characteristics of each septic patient, in order to better choose and monitor the therapy to be tested and the effects to be evaluated.

Pursuing a personalized medicine of septic patients, which also includes the differentiation of types of sepsis and the context in which they develop, the article by Bertozzi et al. gives a detailed description of Neutropenic Enterocolitis (NE) and its frequent association with sepsis [10]. NE is an acute alteration of the intestinal mucosa, mainly of the colon, which develops in immunocompromised patients with a history of cancer and chemotherapy in the previous month, particularly treated with those agents that cause mucositis such as taxanes. This condition has an extremely high lethality rate (79% of patients with histologically confirmed NE died after a median survival of 1 day) and septicemia occurs frequently. The demonstration of an altered intestinal mucosal barrier would seem to support the hypothesis of translocation as a prerequisite for subsequent bacteremia, sepsis, and multi-organ failure.

The study by Perrotta et al. describes the case of a patient affected by thrombotic thrombocytopenic purpura (TTP) admitted to an ICU after developing a hospital-acquired SARS-CoV-2 interstitial pneumonia, further complicated by a K. Pneumoniae NDM sepsis [11]. The antibiotic treatment was effective not only for the treatment of sepsis, but also for intestinal decolonization. This is the first report in literature on intestinal decontamination using the ceftazidime/avibactam + aztreonam combination as treatment for Klebsiella pneumoniae NDM sepsis. This article allows a reflection on the risk of bacteremia related to intestinal colonization with KPC-Kp and on the possibility that a specific acquired condition such as SARS-CoV-2 infection may increase this risk, either through a direct mechanism of infection at intestinal level, either following the use of immuno-modulatory therapies, both capable of increasing bacterial translocation in the intestinal tract.

On this connection, again with a view to a personalized medicine that allows managing the infection in the context of the patient’s singularity, the study by Murri et al. acquires relevance [12]. It took into consideration the management of candidemia and the frequent overtreatment of this condition. Indeed, overtreatment may be associated with several disadvantages: possible increase in antifungal resistance, drugs-related adverse events and high costs. The intervention of a stewardship program associated with a biomarker such as beta-D-glucan has been shown to be effective in reducing the excessive duration of treatment without impacting length of hospitalization or mortality.

Another thriving line of research concerns diagnostic tools for an early and accurate diagnosis of sepsis, taking into account not only the type of infection and the characteristics of host, but also the context in which the patient is being managed.

Covino et al. retrospectively analyzed a monocentric cohort of patients presenting to ED with fever, over a 10-year period, and subsequently hospitalized [13]. The aim of this study was to ascertain whether PCT determination at ED could improve patient’s prognosis with respect to those with no PCT assessment. As main result, in the whole sample of 12.062 evaluated patients, the PCT-guided management was not associated with a better outcome. However, two subgroups of patients showed a clinical benefit from this approach: those who received a final diagnosis of bloodstream infection and those with a qSOFA ≥ 2. At a closer look, this work underlines the importance of PCT in the management of the septic patient, rather than in its early diagnosis.

Even though literature data recommend the use prediction scores to early recognize those patients at risk for sepsis and poor in-hospital outcome, there is still a significant burden of uncertainty on the optimal prognostic score to be used (e.g. qSOFA, SOFA, SIRS, EWS). Sozio and Colleagues focused their research on the most accurate predictor of in-hospital mortality outcome in septic patients presenting to the ED [14]. Their retrospective monocentric study included 1014 patients admitted to two ED in Tuscany, Italy, for suspected sepsis. Among them the diagnosis of sepsis was confirmed in 651 patients, while 363 received an alternative diagnosis. A Bayesian mean multivariate logistic regression model identified septic shock and positive qSOFA as independent risk factors for in-hospital mortality while hyperthermia was a protective factor. In other words, the absence of fever could identify sicker patients who are not able to properly respond to infection (anergy), thus at higher risk of mortality.

Shifting the gaze from the ED setting to that of Internal Medicine departments, the management of patients with a septic state and a bloodstream infection still represents a challenge for the heterogeneity of the population and the scarcity of literature data on the optimal management.

Our research group focused on the prognostic accuracy of delta-PCT (a reduction of PCT > 50% after 48 h, >75% after 72 h, and >85% after 96 h) in predicting mortality of Internal Medicine patients with microbiological identified sepsis [15]. In a sample of 80 patients with at least two available PCT determinations, those patients with Delta-PCT showed a significantly higher proportion of survival both at 28-days and 90-days. Delta-PCT can therefore be used to predict the prognosis of septic patients admitted to internal medicine wards.

The possible application of new diagnostic markers was evaluated by Piccioni et al. who reviewed the current literature on the use of presepsin and proadrenomedullin in the setting of ED [16,17]. Presepsin is a fragment of the soluble form of CD14 (sCD14), after being cleaved by plasma cathepsin D; it contributes to the activation of the innate immune response. Proadrenomedullin derives from the degradation of adrenomedullin into a fragment of 48-amino acids; it is mainly produced by endothelium and smooth muscle cells and exerts its effects on vasodilatation, bronchodilatation, promoting diuresis and myocardial contractility. Levels of these two peptides rise during bloodstream infections, thus they have been proposed in the setting of early identification of septic and critical patients. However, their ability to add significant information compared to those given by PCT is still matter of debate. In any case, given that a single biomarker cannot give an unfailing and absolute answer in the setting of prognostication [9], expanding diagnostic possibilities is warranted.

Similarly, Spoto and Colleagues tested the diagnostic accuracy and prognostic validity of neutrophil-to-lymphocyte (NLR) and platelet-to-lymphocyte (PLR) ratios in comparison with other biomarkers of sepsis in non-ICU wards [18]. They found them good, rapid and cheap biomarkers to help clinician in the identification and prognostic stratification of patients with sepsis.

In summary, the field of sepsis is exceptionally diverse and it is a rapidly growing area of research and development.

We, the Co-Guest-Editors of this Special Issue, are thankful to all the authors and reviewers who have contributed to this issue by sharing their knowledge, findings and time.
